# The organisational climate of NHS Early Intervention Services (EIS) for psychosis: a qualitative analysis

**DOI:** 10.1186/s12913-022-07790-0

**Published:** 2022-04-15

**Authors:** Francis Lammas, Alexandria Phillips, Sue Dopson, Eileen Joyce, Emese Csipke, Til Wykes

**Affiliations:** 1 Institute of Psychiatry, Psychology and Neuroscience, Kings College London, De Crespigny Park, London, SE5 8AF UK; 2 Saïd Business School, University of Oxford, Park End St, Oxford, OX1 1HP UK; 3 The Institute of Neurology, University College London, 12 Queen Square, London, WC1N 3BG UK

**Keywords:** Organisational climate, Psychosis, Early intervention, Clinical teams, Cognitive remediation, Psychotherapy

## Abstract

**Background:**

Cognitive remediation (CR) therapy for psychosis significantly improves recovery but is yet to be widely implemented in NHS services. It is likely to be of value at the earliest stages of psychosis development – at the first episode. Organisational climate is one factor likely to affect implementation into Early Intervention Services (EIS), which serve those experiencing first episode psychosis. We aimed to understand the organisational climate within UK NHS Early Intervention for Psychosis (EIP) services and the barriers and facilitators for the introduction of CR.

**Methods:**

We conducted semi structured interviews with 42 EIS members of four teams in four NHS Trusts. Thematic analysis was used to analyse the data.

**Results:**

There were differences between teams, including leadership style, involvement in decision making and willingness to adopt CR. Resource shortages were considered the main barrier for implementation across all teams. The evidence base behind CR and the recognition of there being a clinical need was seen as the main facilitator. Teams with more democratic leadership, and knowledge of both the evidence base and need for CR, may feel better able to successfully incorporate it into their service.

**Conclusion:**

Despite enthusiasm for novel treatments, EIS teams are limited by their resources. An understanding of the local organisational variables can help teams establish a culture that values innovation. Clear communication of the evidence base for CR is key to help enable staff to implement novel treatments successfully despite these limited resources and time pressures.

**Supplementary Information:**

The online version contains supplementary material available at 10.1186/s12913-022-07790-0.

## Background

Cognitive difficulties at the onset of psychosis, or in those who have a longer illness course, significantly limit functional and clinical outcomes up to 4 years later [1, 2]. Meta-analyses of a novel therapy, Cognitive Remediation (CR), demonstrate significant benefits for global cognition and functioning, particularly when offered alongside other psychiatric care [3–5] and service users also value these benefits [6]. CR is a psychological intervention targeting cognitive dysfunction that aims to improve everyday functioning through increases in a) metacognitive awareness about one’s own strengths and weaknesses as well as thinking skills and their impact on everyday behaviour, and b) metacognitive regulation of that behaviour [7]. CR is included in guidance in Scotland [8], in England [9] and many other countries. CR might improve longer term recovery problems specifically in those at the earliest phase of psychosis. However, there is a gap between efficacy and effectiveness when experimental treatments are deployed into services without clear implementation guidelines and an understanding of the service context.

To bridge this gap, we need to understand the organisational context in which implementation is due to occur [10]. One factor likely to affect both implementation and clinical outcomes is organisational climate. This is the culture in which services operate and is known to help, or hinder, the implementation of new interventions within healthcare systems [11, 12].

The Department of Health established Early Intervention Services (EIS) services from 2001 onwards as specialist NHS services providing treatment and support for people with a first diagnosis of psychosis, (schizophrenia and related disorders) in adults (18 years and older). In 2016 an independent Mental Health Taskforce published their report “*The Five Year Forward View for Mental Health*” [13] which had two implications for the delivery of EIS and informed NICE (National Institute for Health and Care Excellence) Guidelines. The first (NICE recommendation 1.3.1.1.) was that EIS should not have an upper age limit (at that time age 35) and the second (1.3.1.2.) called for rapid assessment of people presenting to services with a possible psychotic illness. Both recommendations increased case coordinators workload, especially as other guidance published at the same time introduced access and waiting time standards which required that “*more than 50% of people experiencing first episode psychosis will be treated with a NICE-approved care package within two weeks of referral*” [14]. Although EIS service staff were known to be enthusiastic about adding novel treatments, this might be a challenge too far in light of simultaneous, multiple recommendations that demand more of existing staff.

This study examines the organisational climate of four NHS EIS services. It uses qualitative, semi-structured interviews to investigate the barriers to, and facilitators of, the potential implementation of a novel therapy, CR, into these services. The results add to our understanding of the factors affecting implementation of treatments into EIP services and also the potential for this treatment to be implemented in other services.

## Methods

### Design

This is a cross-sectional study using qualitative methodology and a comparative case study approach [15, 16]. The study involved interviews with staff members in four EIS teams to generate themes about the local organisational climate and factors associated with implementation. These themes were then compared across the teams to understand shared or different barriers and facilitators to implementation.

### Participants

Four EIS sites were recruited based on their future involvement in a large CR implementation trial (ECLIPSE Trial) and their representativeness of different working environments – urban inner city (1), urban (1), mixed urban/suburban (1), and suburban (1). Team participants were aware of the intervention but had no experience of it. We aimed to interview ten participants per team, including a mix of professional roles. EIS teams typically consist of team leaders of any profession, community psychiatric nurses, social workers, clinical psychologists, occupational therapists, psychiatrists and ‘health care assistant’ grade staff to support the other professionals. We aimed to get a representative mix, there were no exclusion criteria.

Potential participants were contacted individually or through the team. All participants gave written consent after being provided with details of the research and were assured of confidentiality and anonymity. Ethical approval was provided by King’s College London (ref. number LRS15/161724).

### Interview schedule

The semi-structured interview schedule was based on a protocol used by Dopson and Fitzgerald [17], exploring barriers and facilitators of implementation efforts in complex health settings. It was designed to last approximately 30–60 min. The topics covered participants’ experience of the organisational climate and challenges of implementation including: (1) Team working and culture; (2) Team attributes and attitudes toward change; (3) Individual attributes and attitudes toward change; (4) Views on the implementation of the proposed intervention (CR), and; (5) Views on their NHS Trust as an organisation. See Additional file 1 for the full Interview Schedule.

### Data collection and analysis

Researchers, two women and a man, with Master’s level backgrounds in psychology were trained in research procedures, interview techniques, the use of NVIVO, a software for qualitative data management. Analysis and interpretation was supplemented by senior clinical psychologists and a psychiatrist. Participants did not know the researchers prior to the study, and they were completely independent of the trusts and teams involved. The study was explained to participants in the weeks prior to the study, and again as part of the consent procedures. Interviews were carried out at the team base, were audio recorded and transcribed. No one else was present during interview apart from participant and researcher. Data were analysed using inductive thematic qualitative methods described by Braun and Clarke and data analysis followed their six-phase process [18]. Two raters independently familiarised themselves with the data and then generated initial codes leading to categorisation into primary and secondary themes. The researchers discussed their independent analysis and re-interrogated the data to agree and define a final coding framework. In line with a comparative case study design, this process was carried out separately for each team to contrast the similarities and differences between teams. In order to explore the microclimates of each team, results are reported by team in the following sections: organisational climate, facilitators and barriers.

## Results

### Participants

Forty four potential participants were approached to take part, all but two consented. The 42 participants recruited represented different staff roles from the selected EIP teams (see Table 1). Three teams (A, B & D) reported longer length of employment on their wards (4.60–6.77 years) compared to team C (mean = 3.18 years), but data was not collected on team members who did not consent to take part. A report was sent for comment to participants post-analysis, although no comments were returned to the research team. Quotes from the interviews are used here to illustrate our findings, with participants identified only by an assigned number, professional status to protect participant’s identity.

**Table 1:** Number of participants by disciplinary background

	Occupation	Participants	Mean years in service (range)
Team A	Nurse ^a^	4	9.5 (8.0–12.0)
*n* = 11	Psychologist	1	3.0 (n/a)
Mixed urban/suburban	Occupational Therapist	1	1.5 (n/a)
	Psychiatrist	2	4.0 (1.0–7.0)
	Support Worker	3	8.3 (4.0–12.0)
Sub-total		11	6.7
Team B	Nurse ^a^	3	4.0 (0.8–8.0)
*n* = 10	Psychologist	3	1.8 (0.6–4.0)
suburban	Occupational Therapist	1	9.0 (n/a)
	Psychiatrist	1	6.5 (n/a)
	Support Worker	1	8.0 (n/a)
	Other	1	7.0 (n/a)
Sub-total		10	4.8
Team C	Nurse	3	.96 (0.3–2.0)
*n* = 11	Psychologist	4	0.5 (0.3–1.0)
Urban/inner city	Occupational Therapist ^a^	1	5.0 (n/a)
	Social Worker	1	10.0 (n/a)
	Other	2	12
Sub-total		11	3.2
Team D	Nurse	2	6.0 (5.0–7.0)
*n* = 10	Psychologist	1	0.2
urban	Occupational Therapist ^a^	3	3.5 (0.3–9.0)
	Psychiatrist	2	3.7 (0.3–7.0)
	Support Worker	1	–
	Social Worker	1	10.5 (n/a)
Sub-total		10	4.5
Total		*n* = 42	4.6

‘Other’ comprised of two administrative staff and a legal service lead; ^a^ inclusive of team leader

### Analysis

A summary of the main themes and subthemes is presented in Table 2.

**Table 2:** Summary of main themes and subthemes across the teams

Primary Themes	Secondary Themes
1.0 Organisational climate	*1.1 Multidisciplinary Working*
	*1.2 Supportive Culture* *1.3 Flattened Hierarchy* *1.4 Adaptive yet Uncertain*
2.0 Facilitators to Implementations	*2.1 Adequate resourcing and training* *2.2 Evidence Based Therapy* *2.3 Clear rational and communications* *2.4 Recognition of unique needs and benefits* *2.5Pre-existing research culture*
3.0 Barriers to Implementation	*3.1 Resource shortages*
	*3.2 Insufficient support from leaders* *3.3 Implementing too much change at once* *3.4 Intensity of CR* *3.5 ‘Narrow’ applicability of CR*

Not all secondary themes were raised by each team

#### Organisational climate Team A (11 participants)

Team A is a mixed urban/suburban based EIP that serves residents in the local borough. The trust has two EIP teams who share senior managers. Participants reported there was a low staff turnover, noted by participants to be an indicator of good functioning and helpful for maintaining the team’s ethos and goals for early intervention.

*There's now a small group of us that have been around for a number of years that really carry the ethos of what it's all about, and as we grow, and we encourage new people in.* (5)

All participants had positive comments, describing the team culture as inclusive, adaptive, supportive and service-user and research focused.

*It’s a very inclusive team, a very supportive team, we've got a shared goal in terms of having really nice, clear guidelines about what's the sort of work we should be doing which I think has helped focus us.* (5)

Participants also praised the level of group involvement in decision making and leadership. This was seen to be entirely beneficial.

*The culture of this team is, I’d say it’s more of a flattened hierarchy than a lot of other teams, as in when they have the clinical meeting everyone attends, you know, regardless of your band and everyone's input is just as valid.* (10)

### Facilitators for implementation

It was very important for all participants to understand both the rationale and need for CR.

*The treatments we have often don't work or I mean negative symptoms of schizophrenia where there's just a lack of ability to do things, a lack of motivation, blunting of enjoyment, these are the things that our medications can't really treat… these are areas that therapy like that might be able to help with.* (8)

An intervention with an established evidence-base was important.

*I think that's a key, and if they knew the evidence base for… I think people would be much more willing, really willing, as well as having a better understanding and being able to explain it really well.* (6)

### Barriers to implementation

Concern about the computerized nature of CR and the intensity of sessions was raised by some who thought it would be difficult to secure the equipment, time and space to deliver three sessions per week.

*But yes, it would just be about timing, wouldn't it? Because if it was somebody on my caseload that I was doing this with, then I wouldn't be able to do something else with them.* (5)

Some staff also noted the ongoing pressures on staff time and that any new intervention could be an additional pressure’

*A lot of people would embrace it, some people would probably resist it, not on the grounds that they think anything’s wrong with it, it’s just that it’s another pressure.* (10)

#### Team B (10 participants)

##### Organisational climate

Team B is a suburban based EIP that serves residents in the local borough. The trust has multiple EIP teams which are managed separately. Recently, more psychologists had been employed which was seen as a positive change.

*In terms of the cohesiveness, I have a feeling that it is cohesive. The team has gone through changes in relation to psychology especially… So the team have had to kind of adjust to that and I think they've really been able to integrate us quite nicely and openly in to the team.* (19)

All staff had positive comments about the culture, including that it was inclusive, dynamic, fun, welcoming and supportive. There was a low staff turnover which, like other teams, was thought an indicator of good functioning.

*I think everyone's been here for more than three years […] Which is really good, it certainly helps in terms of team cohesion, we don't have many issues with that and we always get on quite well.* (22)

Only one participant disagreed, noting difficulties with clashing personalities from their perspective as a temporary worker.

*It’s kind of quite interesting being an outsider …. because you can, it’s that kind of treadmill, when you see these camps within the teams and, you know… it’s the one-upmanship I think, played out for the benefit of colleagues and the manager, which is kind of predictable and sad and time-wasting.* (16)

The team had recently undergone a change in leadership, from trust management of all EIP teams to a local clinical lead for the borough. This was viewed positively and the team leader retained their role throughout this time and was spoken of positively by staff who felt there was a flattened hierarchical system facilitated by effective leadership.

*It's a fairly happy team and it seems fairly functional. [Name] as a consultant I think plays a big part in that, being quite approachable, containing of peoples’ anxieties, flexible and available and what's the word, they kind of call it a flattened hierarchy. So …[the consultant is] definitely the boss… but at the same time [the consultant is] pretty diplomatic about things and include and trying to get everybody involved.* (17)

##### Facilitators of implementation

The potential benefit of CR was very appealing, team members felt that trying it themselves or seeing first-hand the benefits was key to understanding its utility and therefore increasing the likelihood of successful adoption:

*I guess they're also happy if it just makes common sense, you know, if they've kind of had some firsthand experience, then it helps.* (17)

It was thought vital to have effective communication on the need for CR. This covered information on the change itself, team feedback that helped the team feel heard and recognized would mean they would be more willing to implement the change.

*Getting clinical feedback from us, more openly say why the change is being thought about, getting ideas from the clinical level about how the change could be implemented, what type of change would be most useful and how it could be monitored, so that the staff are involved earlier rather than just at the point of enacting the change.* (19)

There was a real sense of the team’s affinity with innovation. If the team felt involved in the process, understood the benefits of the intervention, and could foster its adoption from within, this would produce successful adoption.

*Sometimes someone from outside coming and saying I want your patients, this is what we're doing, isn’t as effective as people from within trying to develop an idea. I think people quite like championing and making it their baby and then making it happen.* (20)

##### Barriers to implementation

There was concern about the intensity and frequency of CR sessions due to pressures on staff time and service user engagement. Many suggested that given limited resources more staff would be needed to implement a new intervention, and that new staff would also require more space.

*we don't have a desk or we don't have rooms… I think then there's kind of like, you can get hostile against that, you know, you push against it because you're so angry about all the other stuff.* (17)

Some staff highlighted a context of continuous change within the team and trust which felt difficult to manage. Changes in management and working practices alongside the introduction of new interventions felt overwhelming. For respondents, change was more readily accepted when it was standalone and therefore staff could give their full effort and focus to the change.

*I think having one area that's changing at a time and completely unrealistic to expect that, that's ever to be the case […] It would be, so you can really focus your efforts and you can do it properly and you can make sure that's the priority.* (20)

#### Team C (11 participants)

##### Organisational climate

This is a large inner-city service with more than 20 team members. In contrast to other teams, there was a high staff turnover, noted by members as contributing towards feelings of instability. Most staff felt the culture had been fragmented by poor change management and needed improving. As a result, participants often described themselves as under pressure and resistant to change.

*I think there is a sense there that we're swimming against the tide really* (25)

*There is a limit to the amount of change that an organisation can experience at any one point in time I think.* (27)

Despite having a protocol that called for collective decision making, many participants criticised the decision-making process and gave examples of when they felt unable to contribute or have their views genuinely considered in discussions.

*‘A couple of weeks ago we had a really long, difficult disagreement between the team. Well in fact it was just one against everyone else really about whether we should take this person or not, and that was – I don't think anyone was happy with the resolution or the way that it was resolved. It was just kind of that we were overruled by the minority, which was one person’* (29)

Participants also highlighted problems that may arise if senior staff disagree with each other.

*‘I think sometimes between our team leaders, as almost the mum and dad of the team... Sometimes they can kind of have conflicting views... Which then is not great for the rest of the team because if mum and dad aren't agreeing, then the kids are just running riot, and that's how it feels sometimes.’ (31)*

Only one participant disagreed with this sentiment and described the team as very open and supportive.

*‘I think in a way we all still come together and we do support one another, which I think is really good, so it’s a good place, it’s a nice place to be’ (32)*

##### Facilitators for implementation

Many recognized the potential for CR to help service users and considered this a motivating factor for adopting it. Specifically, the computerized aspect of CR was thought to be particularly useful for younger service users, and the potential for CR to reduce the number of sessions needed in subsequent psychological therapies, such as CBT, was thought to beneficial for the entire service.

*The idea that something like this would sort of impact on the amount of sessions of psychology that someone might have, I think something like this is going to be really beneficial. (32)*

*I think there's real potential, I think there's real potential, I think it will appeal to young people, the IT aspect.* (25)

##### Barriers to implementation

Despite staff viewing CR as beneficial, many felt they could not implement it into their team because of current resource shortages, time constraints and added pressures that generally accompany change.

*There’s many different approaches that are being introduced at the EI team… when people do the training – I've done some recently – the motivation to engage in that is very high. When you come back to your team and you're looking at your caseload and you're trying to figure out whether you can commit time to do this, it's a very different kettle of fish* (26)

One team member said they couldn’t see the need for CR with their clients, and if they had enough time they would prefer to train in a therapy with wider suitability.

*It seems the more I heard about this the more I was unsure about whether my clients would be suitable for it, because a lot of my clients are quite high-functioning… It wouldn't be high on my priority list for training to do, I’d want to do family intervention and CPT, really mindfulness or some other training that I can see having a broader suitability than this* (29)

#### Team D (10 participants)

##### Organisational climate

This urban team was located close to clients so staff were able to spend more time at the team base. This increased opportunities for team working and discussion, good functioning and cohesiveness.

*Yes, so I mean we're very lucky in ... (City) because, you know, the city is so accessible and that means that the team do spend a lot of time together at the team base… Yes, so I think the net effect on the team culture is that we're better able to achieve a greater sense of whole team approach to managing the caseload. (38)*

Many team members noted the importance of multidisciplinary working and how helpful this was in providing specialised care. In particular, the unusually high amount of medically trained professionals in the team, and how helpful this was for managing risk and physical health:

*We've got an awful lot of medical kind of angle coming in, which means we are getting quite good at physical health checks and making sure that we're keeping an eye on those targets… they often support you when you have a new assessment, so it’s a really big advantage having that many kind of medically trained people in the team. (41)*

All respondents experienced a unified, supportive and welcoming team that worked together and was not hierarchical in nature. Many described the team as unique in its highly supportive culture, with staff members covering each other and able to talk honestly about difficulties and how these factors contribute to high morale.

*I've never worked in a team where we have like buddy systems and that we cover each other, so we help each other out with any sort of problem tasks that need to be done… it definitely helps morale, it helps, you know… if I'm stuck, I do feel I can say this in the meeting. (42)*

Given the close-knit nature of the team, referred to by some as a ‘family’ structure, it was initially hard to integrate new members. Though this did not affect the positive regard for the team.

*Coming in to the team as a newbie, that meant that initially it was quite difficult, because of course if you've got a very established team, people are already working very closely together, those relationships are quite longstanding, and whilst people were very friendly and welcoming, it did feel like it took a little while to really permeate that culture and become part of the team (39)*

Participants all acknowledged the inevitability of change in the NHS but that given their strengths as a cohesive team they could effectively adapt in these circumstances:

*I watched how the team kind of dealt with uncertainty really… what I quite liked about it was that there wasn't this set rigidity of this is how it happens and this is what you do… There was the acknowledgment that this was something that was developing and evolving and that we could contribute in to that and, you know, I felt that my contributions had been heard… So I think the team has, there's quite an organic way of dealing with change. (39)*

##### Facilitators for implementation

Conveying a clear and coherent rationale for the intervention was imperative for successful implementation. The team were committed to driving innovation and all team members conveyed a desire to learn, train, professionally develop and use evidence-based interventions to deliver the best quality service to the community.

*I'm up for that. Yes, I'm always happy to try things. I mean if there's an evidence to it and it works, then yes, why not, you know, I don't see anything wrong with that (35)*

*Absolutely, yes, and everyone's keen to sort of, you know, add another arrow to the bow in terms of their own professional development (38)*

##### Barriers to implementation

There were few barriers to implementation as this team was pro-active in their approach. The only problems were related to feeling overwhelmed with multiple changes and making sure there was adequate staffing to deliver the intervention:

*And because there have been so many changes within the team in terms of the way that we work and the people that we work with, I think it might be that that could continue and that the ball would keep on rolling or it might mean I guess that people get fatigued and overwhelmed with too much change happening at one time and get a bit exhausted… (34)*

##### Differences between teams

Contextual factors that affect team attitude to change which were common across all teams are presented in Table 3.

**Table 3:** Contextual factors affecting team attitude to change

	Facilitating	Inhibiting
Staff team		
Team A	Supportive culture, low staff turnover and emphasis on group working.	Difficulties in managing on-going change and inexperience in delivering psychological interventions.
Team B	Mix of experiences and competences, low staff turnover and strong research culture.	Difficulties in managing on-going change.
Team C	Supportive staff members.	Influx of new staff and fractured culture due to on-going change.
Team D	Supportive culture, enthusiasm for improving patient care and balanced MDT.	Difficulties in managing on-going change.
Team leadership		
Team A	Team meetings and collective discussion.	
Team B	Approachable, available leadership. Well-liked and respected leader.	
Team C	–	Overtly strong and hierarchical leadership. Indecisiveness and reliance on team leader for decision making.
Team D	‘Flattened’ hierarchy in leadership and decision making. Valuing all opinions.	Initial difficulties integrating new team members.
Infrastructure and working practices		
Team A	Adequate personal space.	Lack of staff, room availability and materials to deliver CR.
Team B	–	Lack of space (hot-desking), resources and staff availability.
Team C	–	General resource shortages and time restraints.
Team D	Adequate space central location enabling more group-working.	Need for adequate staffing to deliver CR.
Intervention		
Team A	Communicating evidence of efficacy and clear need	Intensive workload, clack of clarity and confidence, computerized nature of CR.
Team B	Communication of rationale and benefits, personal experience of efficacy	Intensive workload
Team C	Communicating acceptability (e.g. using computer) and benefits to other therapies.	Narrow applicability and not needed for all service users.
Team D	Communicating clear rationale and evidence-base.	

## Discussion

Teams and participants were representative of EIS teams across the UK, reflecting differences in team location, staff turnover and leadership systems. The data suggest that the teams were functioning well overall, albeit under pressure because of new working practices, national policy changes and resource shortages. The teams, as expected, were interested in research and innovation, and keen to consider CR given its evidence-based benefit to service user [4]. However, they were concerned about managing additional pressures. Themes that arose consistently across teams highlighted the importance of flattened hierarchical structures that enable staff to feel supported but able to be involved in decision making effectively despite NHS pressures. Successful implementation is more likely for interventions based on a distinct need and evidence of benefit in teams that were able to overcome resource shortages through democratic management and involvement in implementation planning. Teams that felt supported by senior staff and had shared experiences in decision making, were more willing to take on extra responsibility for the benefit of service users.

### Resources

Limited resources were frequently cited as a hindrance to the adoption of new working practices. Staff were already concerned about staff shortages, poor working space and time and therefore were averse to adding more responsibilities. This has been noted consistently in other mental healthcare settings [19–21]. In this study CR was characterised by many as demanding time and clinical space since service users are recommended to receive two-to-three CR sessions per week.

### Meeting a clinical need

Staff consistently recognised a clinical need for treating cognitive difficulties in psychosis. This need and potential benefit to service users was noted across all teams and was cited as a driver for the successful adoption of CR, despite its resource demands. All teams emphasised the importance of the evidence base, as well as anecdotal first-hand experience of the effectiveness of new interventions. This is supported by the literature in that people embrace change more readily if they believe the outcome to be successful and beneficial [22–24]. Clear communication from team leaders of the rationale and efficacy of CR was cited as vital for implementation to be successful.

### The importance of democratic leadership

Perceived democratic decision making and positive views about the successful implementation of CR were related in our data. Most team leaders adopted a facilitative, consultative management style and encouraged active participation in discussions which seemed effective in planning for, and reflecting on, proposed changes. The teams who shared decision making focused on the best and most pragmatic method of CR delivery in their unique contexts (e.g. who is best to deliver it, how to train people internally). Conversely, the team who saw themselves as having few opportunities for shared consultation and communication had more negative attitudes. The team also felt less involved in decision making and was also more resistant when discussing CR implementation and focused primarily on their poverty of resources. This team maybe less willing to implement CR without additional support.

Individuals in key leadership roles can influence and stimulate innovation pathways, and clinical opinion leaders are frequently found to play an important role in sponsoring evidence-based innovations [25]. While some leaders allowed team members to take an active role in the decision-making process, other teams used team meetings as a forum for secondary consultation only or communicating decisions already made. The active approach seems to promote autonomy and confidence in the participants in this study, igniting the ‘can-do’ attitude to overcome resource issues and take on new work, whereas the latter approach seems to discourage it. A greater reliance on the leader for decision making and problem solving might leave members feeling disempowered and less capable of tackling change or implementing CR themselves.

### A proposed model of organisational climate as a dynamic mediator of implementation success

**Fig. 1:**
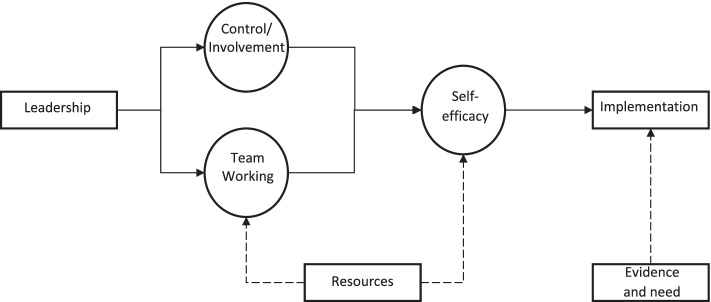
Organisational climate and implementation flowchart. Legend: Note. ‘Leadership’ = Team leadership style; ‘Team working’ = Support and balance of MDT; ‘Control/Involvement’ = Team’s perception of control and involvement in decision making; ‘Resources’ = Supply of resources to support intervention and team; ‘Self-efficacy’ = Confidence, acceptance and belief in successful implementation; Evidence and Need = The recognised need and evidence-base of proposed intervention; ‘Implementation’ = Success of implementation

Our data suggest that successful implementation of an intervention is not through generic (i.e. context free) organisational variables, but rather by considering local conditions that provide the implementation context. Effective leadership helps staff feel in control of their day to day work and being involved in decision making allows staff to ‘own’ it. Good leadership also provides the basis for a well functioning team who truly work with and support each other for the benefit of service users. Both of these things build self efficacy and the confidence in individual team members, allowing them to implement CR effectively. Good leaders also know the importance of communicating with their team the evidence base for particular therapies and the clinical need for it. Combined with adequate resources, these things allow for the effective implementation of therapies, in this case CR. (see Fig. 1). It is critical for leaders and teams to work with these contextual factors to ensure the successful implementation. Future research should look to examine and test the pathways presented in Fig. 1.

### Limitations

The early intervention services involved in this study were all very supportive of and take an active part in research, which may not be reflective of other less research intensive EIS. This may mean that the staff working in these teams are more aware of the value and efficacy of evidence based practice and, in turn, be more supportive of new therapies being introduced to their services. Future studies could involve specifically those services not otherwise involved in research.

### Recommendations

A recent King’s Fund report warns of the length of time it takes to initiate improvements in mental health services [26]. High staff turnover, outdated hierarchical structures, and working pressures, such as frequent change and scarce resources, can dampen staff empowerment and hinder implementation of new interventions [27]. Changing practice fundamentally depends on a set of social processes, including the relation of new evidence to the needs of the local context, discussing and debating evidence with local stakeholders and taking joint decisions about its enactment. The successful adoption of an evidence-based practice depends on all these supportive social processes and requires emphasis by local leadership.

The data from this study call for the identification of clinical opinion leaders who exercise soft and distributed leadership to manage the effective adoption of change and empower staff to take on extra responsibilities for the benefit of the team and service users. Hosting regular meetings, even daily ‘check ins’, helps foster an environment where staff feel valued and heard, and providing a suitable workspace to facilitate this is also vital [28].

Change is often imposed, but the way it is handled by local managers and adopted by local micro-climates can be fundamentally different. Involved managerial staff have more positive views of change [29, 30], so teams given more opportunities to influence decisions should feel more willing to implement any proposed change. Evidence becomes ‘real’ by being enacted in micro clinical settings by local actors and therefore this process must be closely managed by local leaders. A careful management of this collaborative relationship, with a clear time and space for doing so, is likely to yield better change outcomes and effective implementation of CR.

## Conclusion

The findings from this study suggest that the successful implementation of CR, or any novel therapy, is likely to involve highlighting clinical need and creating a ‘can do’ climate through effective and distributed in-house leadership. Engaging all team members in the implementation process through collaborative and consultative decision making can stimulate a flattened hierarchical structure, empowering staff to overcome withstanding NHS pressures to better deliver evidence-based care.

## Supplementary Information

**Additional file 1.** Supplementary materials.

## Data Availability

The dataset analysed during the current study is available from the corresponding author on reasonable request.

## Abbreviations

CR: Cognitive Remediation
EIS: Early Intervention Services
NICE: National Institute of Health and Care Excellence
