# Supplementary material for: The organisational climate of NHS Early Intervention Services (EIS) for psychosis: a qualitative analysis
Source: BMC Health Serv Res. 2022 Apr 15;22:509. doi: 10.1186/s12913-022-07790-0 (PMC9013142; doi:10.1186/s12913-022-07790-0)
Supplement: Supplementary file 1 — Additional file 1. Supplementary materials. [file 12913_2022_7790_MOESM1_ESM.docx]

**ADDITIONAL FILE 1/SUPPLEMENTARY MATERIALS**

**Eclipse WP1 Study 4 Interview Schedule**

Explain Study: Study Team: Confidentiality and anonymity: Sign consent forms

**Name (affirm anonymisation) and position**: Details of role: Length of time on team/in Trust

**(1) Team working**

**(a) Can you describe the team to me?**

*Numbers: Roles: Relationships: Tasks:*

*Culture: Inward/outward looking (eg. individuals' own modes of operating, their beliefs, priorities, values v. focusing more on strategic, performance-based objectives)*

**(b) How do decisions get made at the level of the team?**
*Views on what works well/less well: How are decisions shaped?*

**(c) How do you monitor progress currently?** (eg. progress of team development, achievements)
 *Strengths and weakness of the process?*

**(2) Staff attributes and attitudes toward change** (cohesion, stress, authority, openness to change)

**(a) How does the team respond to change?**
*Describe process (probe for any recent change if necessary):
Barriers: - What can stop change happening? (would a new intervention be a burden or cause additional stress)
Facilitators: - Incentives: Leadership: Role of multi professional groups*

**(b) Does the type or source of the proposed change make a difference?***Familiar/unfamiliar (eg. new and different types of therapy/interventions developed by researchers; Team/individuals' competence more/less important than approach)
Importance of internal or external initiation (eg if it was required by the Trust/or was adopted by NICE)*

**(c) How were team leaders involved in the implementation/change you described?**

*How much and at what levels(organisation, team level etc.)?*

*Did they help or hinder implementation?*

**(d) If you had to provide a metaphor or image to describe the team, what would it be?**

**(e) What do you anticipate helping/blocking future change at the level of the team?**

*If you could change anything in the context or in the team that would help innovations get adopted, what would it be?*

**(3) Individual attributes and attitudes toward change** (*willingness to change, motivation for work, response to pressure to change, trust in leadership, perceived authority)*

**(a) How do you feel generally about using new interventions with your clients?**

**(b) Is there anything which would make you more likely to try a new intervention?**

*(eg If it was being used by colleagues who were happy with it; if the leader was supportive of the new intervention; degree of work involved)*

**(4) Views on proposed intervention (acceptability, sustainability)**

**(a) What do you know about the intervention planned as part of the research project?**

*description; motivation for intervention*

**(b) How successful do you foresee the introduction of CRT to early intervention services for schizophrenia being in improving clinical processes?**

*What do you base this on? (e.g. acceptability to self and others, degree of organisational support)*

**(c) What do you think may be the strengths and weaknesses of your approach locally?**

*Challenges of the CRT implementation? What might help it succeed?*

**(d) How do you think CRT will or will not fit with your daily work in the organization?**

*What changes in clinical practice would you hope to see in future as a result of introducing CRT to early intervention services?*

**(e) How successful do you think introducing CRT to Early Intervention Services will be in changing clinical practice?**

*Influences on change (e.g. national guidelines, experience).*

**(f) How sustainable do you feel these changes will be?**

*Why/why not?*

**(5) Views on the organisation**

**(a) How do you feel about this Trust as an organisation?**

*Organisational values (e.g. informal culture –informal communication, unwritten rules/procedures- , embedded values); policies, procedures, resources; readiness for change, change commitment & change efficiency (effort they are willing to put in, persistence) education, training for staff.* *strengths and weaknesses*

**(b) Is it possible to characterise the trust’s culture?**

*What is like to work here?; Provide a metaphor to describe the culture.*

**(c) How do you think these characteristics influence** **implementation of change?**

*Organizational structures and processes to support implementation? (eg information systems, fidelity tools, feedback, coaching).*

**(6) Background questions for key informants (otherwise go to 7)**

**(a) Could you give me a few key facts about the trust?**

*Brief history of the trust (when was it formed etc.?)*

*Location (number of sites and the geographical distribution)?*

*What is its current size (by workforce, beds and budget)?*

*Recent changes (last five years or so) and explore financial well-being*

**(b) What would you say are the pressures/factors driving trust performance/activity?**

*Business’ plan/strategy: key goals/objectives*

*Explore how performance targets are shaping what is happening in the trust; which ones are driving activity.*

**(c) Does the trust have a research plan/strategy?**

*Summarise key goals/objectives*

**(7) Closing question**

**Any other contributions you would like to make that we have not covered?**

**Thank you**
